# Chemerin and Adiponectin Contribute Reciprocally to Metabolic Syndrome

**DOI:** 10.1371/journal.pone.0034710

**Published:** 2012-04-11

**Authors:** Sang Hui Chu, Mi Kyung Lee, Ki Yong Ahn, Jee-Aee Im, Min Soo Park, Duk-Chul Lee, Justin Y. Jeon, Ji Won Lee

**Affiliations:** 1 Department of Clinical Nursing Science, Yonsei University College of Nursing, Nursing Policy and Research Institute, Biobehavioral Research Center, Seoul, Korea; 2 Department of Sport and Leisure Studies, Yonsei University, Seoul, Korea; 3 Sport and Medicine Research Center, INTOTO Inc., Seoul, Korea; 4 Department of Pediatrics, Clinical Trial Center of Severance Hospital, Yonsei University College of Medicine, Seoul, Korea; 5 Department of Family Medicine, Severance Hospital, Yonsei University College of Medicine, Seoul, Korea; French National Centre for Scientific Research, France

## Abstract

Obesity and metabolic syndrome (MetS) are considered chronic inflammatory states. Chemerin, a novel adipokine, may play an important role in linking MetS and inflammation. We investigated the association of chemerin with inflammatory markers and with characteristics of MetS in apparently healthy overweight and obese adults. We studied 92 adults; 59 men and 33 women whose average body mass index (BMI) was 28.15±5.08 kg/m^2^. Anthropometric parameters, insulin resistance indices, lipid profiles, and inflammatory markers including high sensitivity C-reactive protein (hsCRP), pentraxin 3 (PTX3), adiponectin, and chemerin were measured. Controlling for age, gender, and BMI, serum chemerin level was positively correlated with body fat and serum triglyceride, and negatively correlated with adiponectin and high density lipoprotein cholesterol (HDL- C), and was not correlated with altered hsCRP or PTX3 levels. Among the low, moderate and high chemerin groups, high chemerin individuals are more likely to have lower HDL-C. Conversely, individuals in the low adiponectin group are more likely to have lower HDL-C and show more MetS phenotypic traits than moderate and high adiponectin subjects. To determine the relationships of chemerin and adiponectin to MetS and its components, participants were stratified into four groups based on their chemerin and adiponectin levels (high chemerin/high adiponectin, high chemerin/low adiponectin, low chemerin/high adiponectin, or low chemerin/low adiponectin). Participants who were in the high chemerin/low adiponectin group more likely to have dyslipidemia and MetS (OR: 5.79, 95% CI:1.00–33.70) compared to the other three group. Our findings suggest that chemerin and adiponectin may reciprocally participate in the development of MetS.

## Introduction

Obesity and metabolic syndrome (MetS) are considered to be chronic inflammatory states in which macrophages accumulate in adipose tissue and secrete inflammatory cytokines. Several novel inflammatory markers have been suggested to integrate metabolic and inflammatory signals. The adipose tissue-derived proteins, adipokines are shifted towards the proinflammatory spectrum in obesity, and these hormonal changes may be used as early markers of energy metabolism.

Chemerin is a recently identified adipokine [1] that is highly expressed in liver and adipose tissue and is associated with adiposity, insulin resistance, MetS risk factors, and degree of nonalcoholic fatty liver [2]–[4]. Importantly, chemerin is thought to regulate adipogenesis and metabolic homeostasis in murine and human adipocytes [5]. Additionally, chemerin modulates the innate immune system through its binding to the orphan G-protein coupled receptor, chemokine-like receptor 1 (CMKLR1, ChemR23) and modulates chemotaxis of immature dendritic cells and macrophages [6]–[8]. Recent studies have associated chemerin with several inflammatory markers in obesity and type-2 diabetes [9], [10] Thus, chemerin is considered a candidate in linking inflammation to obesity-related diseases.

In recent studies, abdominal visceral fat accumulation, blood pressure, and lipid profiles were significantly correlated with serum chemerin levels in 173 healthy Korean individuals [11], and a strong relationship between chemerin and key parameters of MetS has been reported in various populations [2], [4], [12]. However, the role of chemerin as a potential causative link between inflammation and MetS in overweight and obese Asian people has not been studied.

The purpose of the current study was to: 1) investigate associations between chemerin and MetS diagnostic parameters, and serum levels of inflammatory and anti-inflammatory markers including high sensitivity C- reactive protein (hsCRP), pentraxin 3 (PTX3), and adiponectin; and 2) determine whether serum chemerin is a novel predictive marker for MetS in overweight and obese Korean adults.

## Methods

This study was approved by the Institutional Ethics Review Board at Severance Hospital and conducted according to the principles expressed in the Declaration of Helsinki. Written informed consent was obtained from all participants.

### Subjects

We enrolled 92 healthy Korean adults whose body mass indices (BMI) were greater than 23 kg/m^2^. None of the subjects had a previous diagnosis of diabetes, moderate to severe hypertension (resting BP>170/100 mmHg), other cardiovascular disease, chronic renal disease, nor acute infectious disease within the prior 6 months. All subjects were classified according to the modified National Cholesterol Education Program (NCEP) criteria for MetS [13], and were diagnosed as MetS patients if three or more of the following criteria were met: 1) waist circumference (WC) over 90 cm in men and over 80 cm in women (for Asians); 2) systolic blood pressure ≥130 mmHg or diastolic pressure ≥85 mmHg; 3) triglyceride ≥150 mg/dl; 4) high-density lipoprotein cholesterol (HDL-C) <40 mg/dl for men, <50 mg/dl for women; or 5) fasting glucose ≥110 mg/dl. Subject characteristics are summarized in Table 1.

**Table 1:** Clinical characteristics of study subjects.

Parameter	Total (n = 92)	Male (n = 59)	Female (n = 33)	p-value
Age	29.58(5.83)	30.0(5.61)	28.82(6.23)	.354
Body mass index (kg/m^2^)	28.15(5.08)	28.72(4.79)	27.14(5.49)	.155
Waist circumference (cm)	94.75(12.67)	97.82(11.53)	89.26(12.93)	.002
Body fat (%)	30.13(8.61)	26.78(7.29)	36.12(7.52)	<.001
SBP (mmHg)	130.7(16.6)	135.2(15.21)	122.6(16.21)	<.001
DBP (mmHg)	81.92(14.67)	86.11(13.99)	74.42(12.91)	<.001
Glucose (mg/dl)	85.09(11.54)	83.37(10.02)	88.15(13.46)	.175
Insulin (µU/ml)	10.51(7.30)	10.57(7.90)	10.41(6.20)	.760
HOMA-IR	2.28(1.75)	2.25(1.87)	2.34(1.54)	.614
Total cholesterol (mg/dl)	193.8(36.2)	194.9(38.8)	191.9(31.68)	.955
HDL-C (mg/dl)	52.73(12.36)	49.99(11.16)	57.63(13.05)	.004
Triglyceride (mg/dl)	124.5(142.9)	144.8(171.3)	88.15(52.42)	.002
hsCRP (mg/dL)	1.17(1.92)	0.76(1.03)	1.88(2.79)	.228
PTX3 (ng/ml)	0.78(0.81)	0.81(0.82)	0.72(0.81)	.478
Adiponectin (ug/ml)	4.80(2.30)	4.29(2.09)	5.73(2.40)	.001
Chemerin (ng/ml)	100.3(23.5)	102.5(23.1)	96.40(24.04)	.231

Data are given as mean (standard deviation). The Kolmogorov-Smirnov test was used to test the Gaussian distribution of parameters *P-*values were calculated by the Student *t*- test (normally distributed variables; age, Body mass index, waist circumference, body fat, SBP, DBP, total cholesterol, HDL-C, and chemerin) or the nonparametric Wilcoxon rank sum test. (non-normally distributed variables; glucose, insulin, HOMA-IR, triglyceride, hsCRP, PTX3, and adiponectin). SBP: systolic blood pressure, DBP: diastolic blood pressure, HOMA-IR: homeostasis model assessment-insulin resistance, HDL-C: high density lipoprotein–cholesterol, hsCRP: high-sensitivity C-reactive protein, PTX3:pentraxin 3.

### Anthropometric measurement

BMI was calculated with dividing weight by square of height (kg/m^2^). WC was measured at the midpoint between the lower border of the rib cage and the iliac crest. Body fat percentage was measured by bioelectric impedance analysis equipment (Inbody 330, Biospace, Seoul, Korea). Brachial artery blood pressure was measured with a sphygmomanometer with the subject in a sitting position at rest for two minutes.

### Blood collection and biochemical analyses

Blood samples were collected between 7:30 AM to 8:30 AM after overnight fasting. Venous blood was collected, centrifuged, and the separated serum was frozen immediately at −80°C. Serum levels of fasting glucose, total cholesterol (TC), HDL-C and triglycerides were assayed using an ADVIA 1650 Chemistry system (Siemens, Tarrytown, NY, USA). Fasting insulin was assayed via electrochemiluminescence immunoassay using Elecsys 2010 (Roche, Indianapolis, IN, USA). hsCRP was measured using an ADVIA 1650 Chemistry system (Siemens, Tarrytown, NY, USA). Insulin resistance was estimated according to the homeostasis model assessment of insulin resistance (HOMA-IR), (insulin (µIU/ml)×fasting blood glucose (mg/dl)/18)/22.5. Plasma PTX3 levels were measured using a commercially available enzyme-linked immunosorbent assay (ELISA, R&D Systems, Minneapolis, MN, USA), and inter- and intra-assay variability were 5.1±1.0% and 4.0±0.4%. Serum chemerin and adiponectin levels were also measured with an enzyme immunoassay kit (Mesdia, Seoul, Korea), and inter- and intra-assay variability were 11.3±6.0% and 8.4±3.7% for chemerin, and the inter-assay and intra-assay variability was 4.6±1.4% and 4.5±0.6% for adiponectin, respectively.

### Statistical analysis

SAS 9.2(SAS Institute, Cary, NC, USA) was used for statistical analyses. The Student t test or the nonparametric Wilcoxon rank sum test was used to compare baseline characteristics and chemerin levels between men and women. After adjusting for age, gender, and BMI, Pearson's correlation coefficients and partial correlations were calculated to evaluate the relationships between serum chemerin level and glucose metabolism-related parameters, lipid profiles, and inflammatory markers. Participants were divided into three groups based on serum chemerin and adiponectin level (low, moderate, high) respectively and a logistic regression was performed to compare these three groups. To determine the relationships of chemerin and adiponectin to MetS and its components, we assessed the interaction analysis between chemerin and adiponectin for MetS using likelihood-ratio tests comparing regression models. Because the interaction terms of chemerin with adiponectin were significant, participants were stratified into four groups based on their chemerin and adiponectin level (high chemerin/high adiponectin, high chemerin/low adiponectin, low chemerin/high adiponectin, or low chemerin/low adiponectin). ANCOVA was used to determine the differences among the four groups based on chemerin and adiponectin levels. Chi-squared analysis confirmed the relationship between serum chemerin/adiponectin and the diagnosis of the MetS. A logistic regression was used to predict the diagnosis of MetS among four groups. The interaction between chemerin and adiponectin was tested at a significance level of 0.10. Hypothesis testing was two-sided at a significance level of 0.05.

## Results

Table 1 shows the anthropometric and metabolic characteristics of the participants. There was no gender difference in chemerin levels. However, women had significantly higher serum adiponectin levels and significantly lower serum triglyceride levels, compared to men.

Significant correlations were found between chemerin and adiposity, blood pressure, insulin resistance, lipid profiles, hsCRP, and adiponectin; however, there were no significant correlations between chemerin and PTX3 after adjustment for age and gender. Serum chemerin levels were positively correlated with percentage of body fat (r = .281, p<.05) and triglyceride levels (r = .354, p<.05), and were negatively correlated with adiponectin (r = −.336, p<.05) and HDL-C levels(r = −.295, p<.05), but were not correlated with serum hsCRP nor PTX3 after additional adjustment for BMI (Table 2).

**Table 2:** Correlations between chemerin levels and other metabolic parameters (n = 92).

Parameter	Model 1	Model 2
Body mass index (kg/m^2^)	.456*	-
Waist circumference (cm)	.444*	.075
Body fat (%)	.518*	.281*
SBP (mmHg)	.336*	.052
DBP (mmHg)	.331*	.038
Glucose (mg/dl)	.158	.043
Insulin (µU/ml)	.424*	.185
HOMA-IR	.417*	.179
Total cholesterol (mg/dl)	.226*	.174
HDL-C (mg/dl)	−.451*	−.295*
Triglyceride (mg/dl)	.477*	.354*
hsCRP (mg/dL)	.382*	.139
PTX3 (ng/ml)	−.027	.048
Adiponectin (ug/ml)	−.467*	−.336*

Coefficients are calculated using the Pearson correlation model,

* p<0.05 After glucose, insulin, HOMA-IR, triglyceride, hsCRP, PTX3, and adiponectin were log-transformed to improve the skewness of the distribution, Pearson's correlation coefficients were calculated to evaluate the relationship between chemerin levels and metabolic variables.

Model 1. Partial correlation between chemerin and other parameters after adjustments were made for age and gender.

Model 2. Partial correlation between chemerin and other parameters after adjustments were made for age, gender, and BMI.

SBP: systolic blood pressure, DBP: diastolic blood pressure, HOMA-IR: homeostasis model assessment-insulin resistance, HDL-C: high density lipoprotein –cholesterol, hsCRP: high-sensitivity C-reactive protein, PTX3:pentraxin 3.

Logistic regression analyses were performed after participants were categorized according to their chemerin and adiponectin levels (low, moderate, and high serum chemerin and adiponectin levels). Unadjusted logistic regression analyses showed that the risk of MetS increased as chemerin participants with high levels of serum chemerin are more likely to have dyslipidemia with low HDL-C (OR: 15.12, 95% CI: 1.58–144.5) after adjustment for age, gender, and BMI. On the other hand, participants with low levels of serum adiponectin are more likely to have dyslipidemia with low HDL-C (OR: 7.11, 95% CI: 1.02–49.68) and show more MetS phenotype (OR: 19.49, 95% CI:1.63–233.3) after adjustment for age, gender, and BMI (Table 3).

**Table 3:** Risk of having a MetS component relative to chemerin and adiponectin levels.

		Events/No.	Chemerin		Events/No.	adiponectin	
			UnadjustedOR (95% CI)	AdjustedOR (95% CI)		UnadjustedOR (95% CI)	AdjustedOR (95% CI)
WC	Low	18/30	1.00	1.00	29/31	**9.67(1.94–48.28)**	1.73(0.01–20.67)
	Moderate	26/31	**3.46(1.04–11.56)**	0.62(0.10–3.77)	25/31	2.78(0.88–8.79)	2.01(0.28–14.65)
	High	28/31	**6.22(1.54–25.15)**	1.55(0.13–18.94)	18/30	1.00	1.00
BP	Low	9/30	1.00	1.00	21/31	**6.90(2.22–21.42)**	0.93(0.20–4.27)
	Moderate	16/31	2.49(0.87–7.12)	1.09(0.30–4.04)	20/31	**5.97(1.95–18.33)**	2.38(0.60–9.42)
	High	23/31	**6.71(2.19–20.58)**	1.81(0.40–8.19)	7/30	1.00	1.00
Glucose	Low	2/30	1.00	1.00	3/31	**1.50(0.23–9.68)**	2.39(0.16–35.07)
	Moderate	2/31	0.97(0.13–7.33)	0.69(0.08–6.21)	1/31	0.47(0.04–5.44)	0.60(0.04–9.35)
	High	2/31	0.97(0.13–7.33)	0.70(0.06–8.47)	2/30	1.00	1.00
TG	Low	1/30	1.00	1.00	14/31	**9.16(1.84–45.58)**	1.70(0.14–20.23)
	Moderate	7/31	8.46(0.97–73.64)	4.97(0.54–46.08)	6/30	2.53(0.80–7.98)	1.98(0.27–14.41)
	High	12/31	**18.32(2.20–152.7)**	7.30(0.75–70.34)	1/31	1.00	1.00
HDL-C	Low	1/30	1.00	1.00	12/31	**8.84(1.77–44.07)**	**7.11(1.02–49.68)**
	Moderate	5/31	5.58(0.61–50.91)	3.70(0.38–36.33)	6/31	3.36(0.62–18.19)	2.58(0.39–16.86)
	High	14/31	**23.88(2.88–198.0)**	**15.12(1.58–144.5)**	2/30	1.00	1.00
MetS	Low	2/30	1.00	1.00	15/31	**27.19(3.28–225.2)**	**19.49(1.63–233.3)**
	Moderate	7/31	4.08(0.77–21.55)	1.96(0.32–11.87)	9/31	**11.86(1.40–100.7)**	9.90(0.91–107.3)
	High	16/31	**14.94(3.02–73.83)**	6.03(0.97–37.72)	1/30	1.00	1.00

Logistic regression. OR: odds ratio, CI: confidence interval, PTX3:pentraxin 3, Adjusted:adjustment for age, gender, and body mass index.

WC : waist circumference >90 cm in men and >80 cm in women, BP: systolic blood pressure ≥130 mmHg or diastolic pressure ≥85 mmHg, Glucose : ≥110 mg/dl, TG : triglyceride ≥150 mg/dl, HDL-C : high-density lipoprotein cholesterol <40 mg/dl in men and <50 mg/dl in women.

These results indicate a possible reciprocal contribution of chemerin and adiponectin to MetS. After confirming that there was a statistically significant interaction between chemerin and adiponectin, (data not shown) participants were stratified into four groups based on their median levels of chemerin and adiponectin: 1) high chemerin/high adiponectin, 2) high chemerin/low adiponectin, 3) low chemerin/high adiponectin, or 4) low chemerin/low adiponectin, for further analyses. Based on our initial analyses, we anticipated that participants with a high chemerin and low adiponectin would show the worst metabolic profiles. Indeed, this group showed the highest prevalence of MetS compared to that of other three groups. In addition, subjects with high chemerin/low adiponectin showed significant higher triglyceride levels after controlling for age, gender, and BMI (Table 4). To confirm the relative risk of MetS according to chemerin and adiponectin levels, logistic regression was performed. Participants who were in the high chemerin/low adiponectin group still showed the highest relative risk of MetS (OR: 5.79, 95% CI:1.00–33.70) after age, gender, and BMI adjustments (Table 5).

**Table 4:** Anthropometric and metabolic parameters relative to chemerin and adiponectin levels.

	Low chemerin		High chemerin	
	High adiponectin (n = 29)	Low adiponectin (n = 18)	High adiponectin (n = 17)	Low adiponectin (n = 28)
Waist circumference (cm)	94.84(1.00)	93.76(1.18)	95.12(1.25)	95.1(1.07)
Body fat (%)	28.76(0.80)	29.32(0.93)	31.45(0.99)*	31.27(0.85)
SBP (mmHg)	130.8(2.39)	129.5(2.79)	135.3(2.97)	128.6(2.53)
DBP (mmHg)	81.43(1.95)	82.88(2.28)	86.78(2.43)	78.85(2.07)‡
Glucose (mg/dl)	86.95(2.25)	85.78(2.63)	81.39(2.80)	84.96(2.39)
Insulin (µU/ml)	9.53(1.15)	10.43(1.34)	8.47(1.42)	12.82(1.21)‡
HOMA-IR	2.11(0.28)	2.28(0.33)	1.72(0.35)	2.80(0.30)‡
Total cholesterol (mg/dl)	187.2(7.21)	190.4(8.41)	184.3(8.95)	208.7(7.64)‡
HDL-C (mg/dl)	57.74(2.01)	51.18(2.35)*	54.78(2.50)	47.30(2.13)* ‡
Triglyceride (mg/dl)	83.09(27.19)	96.84(31.72)	98.00(33.76)	201.1(28.8)* † ‡
hsCRP (mg/dL)	0.955(0.321)	1.178(0.375)	1.033(0.399)	1.459(0.341)
PTX3 (ng/ml)	0.90(0.16)	0.51(0.19)	1.07(0.20)†	0.66(0.17)
Adiponectin (ug/ml)	6.67(0.30)	3.39(0.35)*	5.51(0.37)* †	3.37(0.31)* ‡
Chemerin (ng/ml)	81.82(2.77)	85.06(3.23)	115.3(3.44)* †	120.2(2.94)* †
Metabolic syndrome n (%)	3(10.3%)	3(16.7%)	2(11.8%)	17(60.7%)^§^

Data are given as mean (standard deviation), adjusted for age, gender, and BMI. ANCOVA was used to determine the differences among four groups based on chemerin and adiponectin levels. ^§^Chi-squared analysis confirmed the relationship between serum chemerin/adiponectin and MetS diagnosis.

* p<0.05 versus low chemerin/high adiponectin,

† p<0.05 versus low chemerin/low adiponectin,

‡ p<0.05 versus high chemerin/high adiponectin, SBP: systolic blood pressure, DBP: diastolic blood pressure, HOMA-IR: homeostasis model assessment-insulin resistance, HDL-C: high-density lipoprotein –cholesterol, hsCRP: high-sensitivity C-reactive protein, PTX3:pentraxin 3.

**Table 5:** Risk of metabolic syndrome relative to chemerin and adiponectin levels.

	Events/No.	Unadjusted OR (95% CI)	Adjusted OR (95% CI)*
Low chemerin/High adiponectin	3/29	1.00	1.00
Low chemerin/Low adiponectin	3/18	1.73(0.31–9.69)	1.10(0.16–7.35)
High chemerin/High adiponectin	2/17	1.16(0.17–7.72)	0.46(0.05–4.16)
High chemerin/Low adiponectin	17/28	**13.39(3.25–55.16)**	**5.79(1.00–33.70)**

Logistic regression. OR: odds ratio, CI: confidence interval, PTX3:pentraxin 3,

* adjusted for age, gender, and BMI.

## Discussion

Altered circulating adipokine levels can be used as early abnormal markers, and may contribute to MetS development. This study addressed the relationship between chemerin, a novel adipokine, and inflammatory and anti-inflammatory markers, and MetS criteria. We found a positive association between circulating chemerin levels and MetS prevalence, and a consistently negative correlation between chemerin and adiponectin levels, the latter of which is known for its protective role in diabetes, MetS, atherosclerosis, and various inflammatory processes [14]. When participants were stratified into four groups based on their relative chemerin and adiponectin levels, participants in the high chemerin/low adiponectin group showed significantly higher triglyceride levels and a greater incidence of MetS than other three groups, after adjusting for age, gender, and adiposity. Moreover, there was a statistically significant interaction between chemerin and adiponectin and logistic regression analysis showed that high chemerin/low adiponectin was an independent risk factor for MetS in the current study. Based on the current observation, we postulate a reciprocal contribution of chemerin and adiponectin to MetS.

Precisely how chemerin and adiponectin reciprocally contribute to the development of MetS is unknown. Although it is clear that elevated serum chemerin levels are associated with obesity or obesity-related diseases, the regulatory mechanisms of chemerin expression are poorly understood. Recent evidence showed that inflammatory cytokines may play a role in chemerin release from adipose tissue. Both IL-1β [15] and TNF-α [16] induce chemerin mRNA expression and secretion from 3T3-L1 adipocytes *in vitro*, and TNF-α increases serum total chemerin levels in wild- type mice but not in TNF receptor superfamily 1a/1b-deficient mice *in vivo* [16]. In the case of adiponectin, TNF-α decreases adiponectin mRNA expression in adipocytes [17], and TNF-α antagonism with etanercept in obese subjects with MetS increases both total and high molecular weight adiponectin [18]. Together, these data suggest that TNF-α regulates the expression of both chemerin and adiponectin, but with opposite effects, in obesity-related inflammation. These findings are supported by the fact that elevated inflammatory cytokine levels and decreased circulating adiponectin are commonly associated with obesity and MetS [14]. In addition, our study revealed the conflicting relationship between chemerin, adiponectin and HDL C which has anti-inflammatory property as well as antiatherogenic functions promoting reverse cholesterol transport [19]. Recent studies investigated the relationship between chemerin expression and peroxisome proliferator-activated receptor γ (PPARγ), a nuclear receptor that is a critical regulator of adipogenesis and adipokine expression. Rosiglitazone, a PPARγ agonist, reduces expression of chemerin selectively in mature adipocytes, both *in vitro* and *in vivo* [20]. Considering that PPARγ is also a positive regulator of adiponectin gene expression and promotes adiponectin synthesis and secretion [21], the inverse relationship between adiponectin and chemerin expression may represent a concomitant metabolic consequence of PPARγ activation. However, consensus has not been reached regarding the role of PPARγ activation in chemerin induction [20], [22]. Further studies are required to answer whether the apparent reciprocal relationship between chemerin and adiponectin is merely a reflection of obesity-related inflammation or is the result of specific regulation by common mediators in adipocytes.

As with the short pentraxin CRP, PTX3, a member of the long pentraxin family, has been suggested to comprise a mechanistic link between chronic low-level inflammation and obesity [23]. However, we found no association between either hsCRP or PTX3, and chemerin levels. This may be because our study participants were relatively healthy obese people without established diabetes or coronary artery disease, and a chronic low-grade inflammatory marker might not be significantly elevated. We cannot exclude the possibility that a larger study population might elucidate subtle relationships between CRP or PTX3 levels, and circulating chemerin levels.

We have confirmed previous reports that increased serum chemerin is associated with components of MetS including elevated TG and lowered HDL-C. However, we did not find any association between chemerin levels and insulin resistance after controlling for age, gender and adiposity. In accordance with our results, Bozaoglu et al. [2], reported that chemerin levels were independently associated with MetS but not with measures of insulin sensitivity or glucose homeostasis in people with normal glucose tolerance. However, several studies have suggested that chemerin represents an independent mechanism linking adipose tissue dysfunction to impaired glucose metabolism and insulin resistance [24], [25]. Recently, Takahashi M et al. demonstrated that insulin sensitivity was enhanced in chemerin(−/−) mice in tissue specific manner and a pivotal transcriptional factor for b-cell function was downregulated in chemerin-deficient islets and a chemerin-ablated b-cell line [26]. The reason for this discrepancy remains to be elucidated.

The cross-sectional nature of our study limits the determination of the temporality or causality, and careful consideration of the low statistical power with small sample sizes and case events will be needed. In addition, we used fasting serum glucose for the detection of diabetes instead of oral glucose tolerance test (OGTT). Although measuring fasting serum glucose is more convenient and reproducible than performing an OGTT, there is a chance that overlooked impaired glucose tolerance might affect the levels of chemerin or adiponectin. This warranted further study. Finally, the chemerin ELISA that we used cannot distinguish prochemerin from chemerin [27]; thus, further studies using methods that can distinguish chemerin variants are needed to define the exact relationship between chemerin and other inflammatory cytokines. In lieu of these limitations, this is the first study to provide new insights into the reciprocal contribution of chemerin and adiponectin to MetS pathogenesis.

In summary, among apparently healthy Korean adults, individuals with high chemerin serum levels coupled with low circulating adiponectin are at significantly increased risk of dyslipidemia and MetS. Further studies will clarify the efficacy of using relative levels of adiponectin and chemerin as a novel combination biomarker for predicting risk of MetS development. This new approach might prove to be a valuable prognostic tool that would complement existing strategies in evaluating a patient's propensity for MetS.
